# Controlling *Alternaria cerealis* MT808477 Tomato Phytopathogen by *Trichoderma harzianum* and Tracking the Plant Physiological Changes

**DOI:** 10.3390/plants10091846

**Published:** 2021-09-06

**Authors:** Ghada Abd-Elmonsef Mahmoud, Mohamed A. Abdel-Sater, Eshraq Al-Amery, Nemmat A. Hussein

**Affiliations:** 1 Botany and Microbiology Department, Faculty of Science, Assiut University, Assiut P.O. Box 71516, Egypt; masater59@yahoo.com (M.A.A.-S.); eshraqalamery@yahoo.com (E.A.-A.); nemmgoda@aun.edu.eg (N.A.H.); 2 Department of Applied Microbiology, Faculty of Applied Sciences, Taiz University, Taiz 6350, Yemen

**Keywords:** *Alternaria cerealis*, *Trichoderma harzianum*, plant defense, antimicrobial activity

## Abstract

Plant responses during the pathogen infection and the pathogen control reflect its strategies to protect its cells. This work represents the *Alternaria cerealis* MT808477 as a phytopathogen causing leaf spot disease in tomatoes. *A. cerealis* was identified morphologically and genetically by 18SrRNA, and its pathogenicity was confirmed by light and scanning electron microscopy. *Trichoderma harzianum* has the ability to control *A. cerealis* MT808477 by stimulating various cell responses during the controlling process. The cell behavior during the biological control process was observed by analyses of total phenol, flavonoids, terpenoids, antioxidant, malondialdehyde and antioxidant enzymes (catalase and peroxidase). The extracts of infected tomato leaves were tested against plant and human pathogenic microorganisms. Results showed that the biological control process activates the defense cell strategies by increasing the plant tolerance, and activation of plant defense systems. The total phenol, flavonoids, terpenoids, antioxidant and malondialdehyde were increased after 48 h. Catalase and peroxidase were increased in infected tomato plants and decreased during the biological control process, reflecting the decrease of cell stress. Leaves extract inhibited the growth of nine plant and human pathogenic microorganisms. Biological control represents a safe and effective solution to phytopathogens that decreases plant cell stress by stimulating various defensive agents.

## 1. Introduction

Tomato (*Solanum lycopersicum* L.) plants are a strategic crop which are full of safe phytochemicals that could prevent major human chronic diseases [1]. Plant pathogenic fungi generate alterations in the plant development stages with various degrees of disease severity depending on the environmental conditions and the plant resistance, causing less productivity, quality, and shelf lifetime, and mycotoxins could reach to the consumers. *Alternaria* is a prevalent fungal genus with distribution as saprophytic and pathogenic species [2,3]. Leaf spot symptoms of *Alternaria* sp. can cause damage in tomatoes at all the growth stages [4]. Phytopathogenic fungi are generally controlled by synthetic fungicides; however using these chemical agents causes many more health and environmental issues due to their harmful effects on human, animal health and the environment. Additionally, excessive usage of these chemicals generates pathogen resistance, which forces researchers to seek natural, safe and effective alternative antimicrobial agents. *Trichoderma* species act as biological control agents that have been reported to suppress important plant diseases and can effectively induce plant defense against various pathogens. *Trichoderma* has the ability to promote plant growth, absorb nutrients, and induce plant defense responses to different biotic and abiotic stresses [5]. The application of *T. harzianum* in biological control showed a significant reduction in disease incidence, with some evidence supporting its ability to induce defense mechanisms in plants [6]. Zghair et al. [7] reported that the biological preparation of *T. harzianum* can effectively reduce the disease intensity of tomato plants by increasing enzymatic antioxidants such as peroxidase, catalase, and polyphenol oxidase. The production of reactive oxygen species (ROS) and the antioxidants enzymes are the main indicator of early plant defense responses encountered by fungal pathogens [8]. One of the main functions of the antioxidant system is to control various biological and physiological processes, including acting as a key intermediate signal to relieve biotic and/or abiotic stress, including in plant growth and development [9]. In protective signal transduction pathways, antioxidants can thus play a very important physiological role as a secondary messenger and also act as one of the first cell responses to invading pathogens associated with damage to cell homeostasis. This leads to the expression and activation of plant defense-related genes via phytoalexin formation, cell wall deposition, and protein-related pathogenesis (PR) proteins [5,10]. Currently, there are numerous reports of plant products providing antibiotic activities against a wide variety of pathogenic microorganisms. Multiple classes of antimicrobial products, including phenolic acids and polyphenols, phenanthrenes, flavonoids, and terpenoids have been described [11,12,13,14]. The production of antimicrobial products by plants after pathogen identification results in the secretion of active antimicrobials to infected sites that may be used in the treatment of human diseases caused by microbes [15]. The purpose of this work is to use the *Trichoderma harzianum* as a biological control to evaluate its ability to reduce leaf spot disease caused by *A. cerealis* MT808477 and inhibit pathogenic microorganisms. We monitored the defense responses of the plant cells by stimulation of many chemical signals that cause a considerable increase in the activity of some chemical defense.

## 2. Results

### 2.1. Pathogenicity of Alternaria cerealis in Tomato Plants

**Figure 1:**
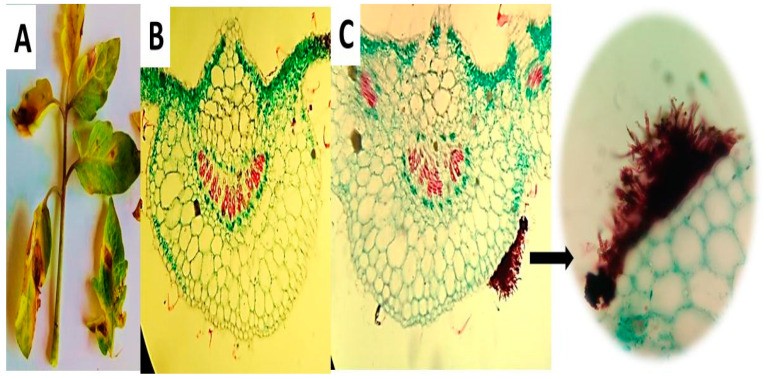
Dark spot lesions generated by *Alternaria* *cerealis* (MT808477) pathogen in tomato leaves (**A**); transverse sections of the healthy leaf region (**B**); and infected leaf region showed brownish fungal conidiophores and mycelia on the leaf surface (**C**) using light microscope (LM).

**Figure 2:**
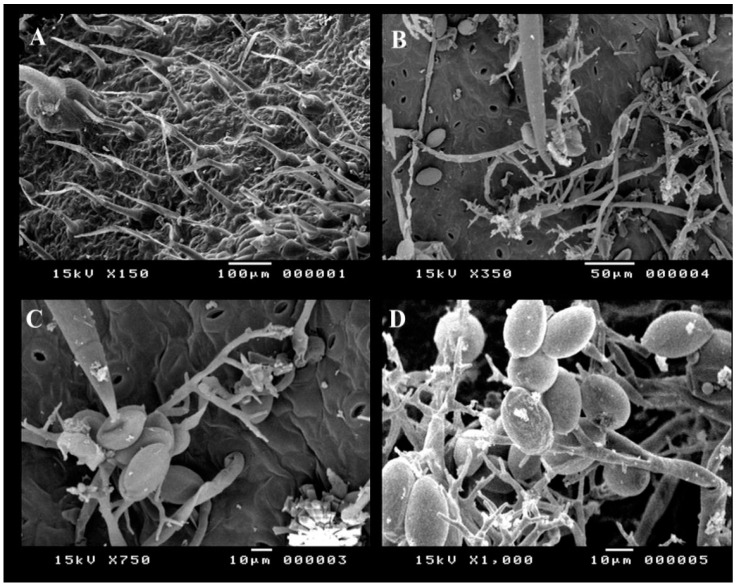
Scanning electron microscopy (SEM) of healthy and infected tomato leaf surfaces; healthy tomato leaf surface (**A**), *A.* *cerealis* (MT808477) conidiophores, mycelia and conidia on the surface of infected tomato leaf (**B**,**C**), and conidiophores bearing lots of *A.* *cerealis* conidia (**D**).

*Alternaria cerealis* (AUMC 14484) was isolated from dark spot lesions in tomato leaves and used to infect healthy plants to confirm its pathogenicity. This work is the first to report *A. cerealis* as a tomato phytopathogen causing dark leaf spot lesions. Infection symptoms in leaves manifested as yellow halos with small brown spots. In later stages of infection, the spots gradually increased in the size and formed dark brown concentric circles. All of the plants infected with *A. cerealis* developed lesions, thereby indicating 100% pathogenicity of the fungus. Transverse sections of the tomato leaves were established to confirm plant infection. Healthy leaf regions were clear, and infected leaf regions showed brownish fungal conidiophores and mycelia on the leaf surface (Figure 1). Healthy and infected tomato leaf surfaces were examined by SEM to confirm infection Healthy tomato leaves showed clear surfaces without infection, whereas infected leaves revealed dark spot lesions with *A. cerealis* mycelia and conidiophores bearing a large number of conidia (Figure 2).

### 2.2. Morphological and Molecular Identification of Alternaria cerealis

**Figure 3:**
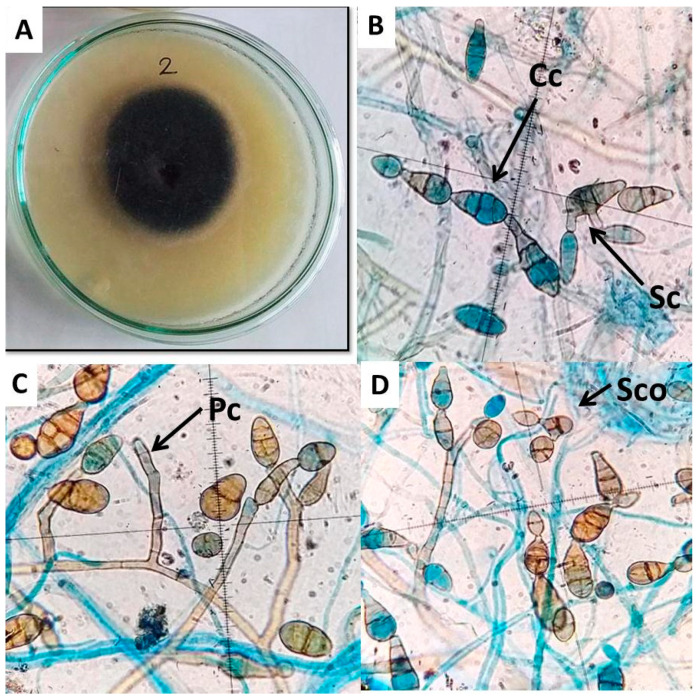
Microscopic feature of *A. cerealis* (MT808477) including; growth on PDA medium after 7 days (**A**); secondary conidiophore (Sc) pears secondary conidia, conidial chain (Cc) (**B**); primary conidiophore (Pc) (**C**); secondary conidia (Sco) (**D**); Scale bars 20 µm.

**Figure 4:**
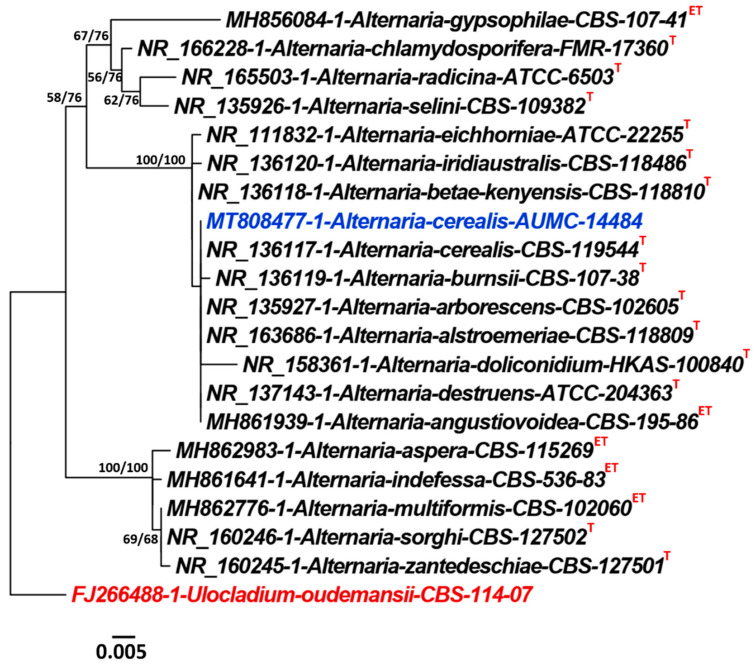
Phylogenetic tree generated from maximum parsimony (MP) analysis based on ITS sequence data of *Alternaria cerealis* AUMC 14484 (MT808477) in blue color associated with other related genes in the ITS gene sequences belonging to *Alternaria*. Bootstrap support values (100 replications) for ML/MP combination equal to or greater than 50% are indicated at the respective nodes. The tree is rooted to *Ulocladium oudemansii* CBS 114.07 as outgroup (in red color). Taxa derived from type materials are indicated with superscripts^(T)^ and that from ex-type specimens with superscripts ^(ET)^.

*Alternariacerealis* (E.G. Simmons & C.F. Hill) AUMC 14484 was identified according to its morphological properties, including colony morphology, color, conidia, conidiophore, and chlamydospore formation, under a light microscope. Colonies of *A.* *cerealis* show dark black mycelia surrounded by a white–gray mycelium in the initial stages of growth. The colonies then turned dark brown in later development stages (Figure 3A). Microscopically, the fungal isolate revealed chains of multicellular smooth-walled pale brown catenulate conidia with short beaks, secondary conidiophores of approximately 50 µm bearing secondary conidia (20–30 µm × 10–12 µm), and pale brown conidiophores ranging in length from 50 µm to 100 µm (Figure 3B–D). ITS sequencing of *A.* *cerealis* AUMC 14484 showed high similarity (>99%) with GenBank accession numbers *Alternaria cerealis* CBS 119544^T^ (Accession no. NR_136117.1), and (100%) with *A. angustiovoidea*, (MH861939.1 = CBS 195.86^T^), *A. destruens* (NR_137143.1 = ATCC 204363^T^), *A. alternata* (MN615420.1 = YZU 191238), *A. alternata* (MN615420.1 = ZTCA11), *A. alternata* (MN481948.1 = A-11), and *A. alternata* (MN402464.1 = SQCM-03) (Figure 4).

### 2.3. Alternaria cerealis Growth Inhibition

**Figure 5:**
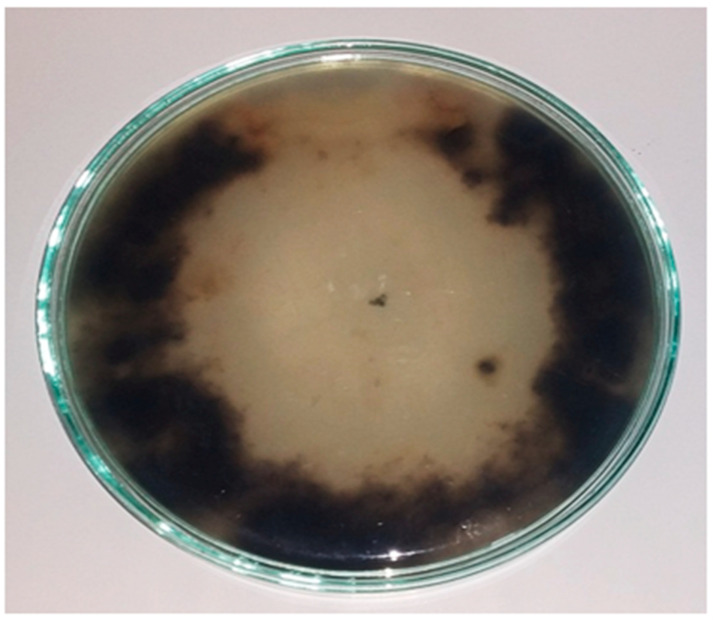
Antagonistic effect between *Alternaria cerealis* (MT808477) tomato (*S. lycopersicum* L.) leaf spot pathogen and saprophytic *Trichoderma harzianum*, showing growth inhibition of *A. cerealis*.

The results indicated that saprophytic *Trichoderma harzianum* showed high antagonistic ability against *A. cerealis* in PDA plates on the fifth day of incubation (Figure 5).

### 2.4. Effect of Infection Period on the Total Phenol Production of Tomato Leaves during the Biological Control

**Figure 6:**
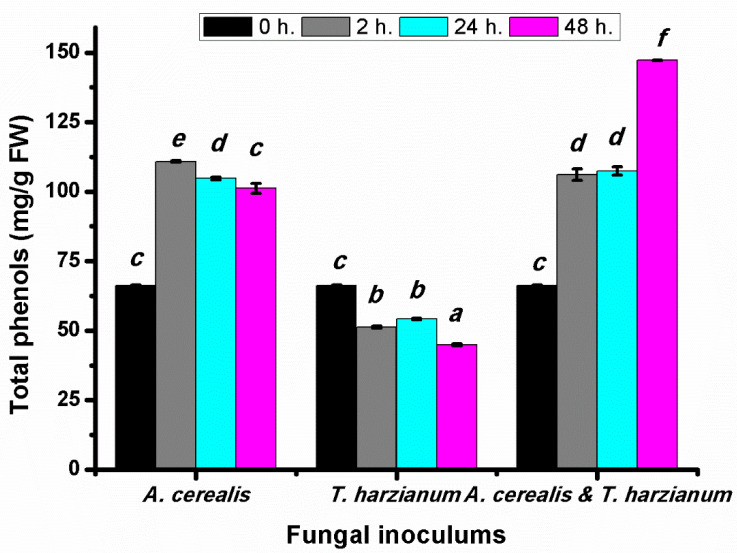
Total phenols (mg/g FW) of tomato leaves (*S. lycopersicum* L.) under the effect of pathogen (*Alternaria cerealis* MT808477), and pathogen inhibitor (*Trichoderma harizainum*) after 0, 2, 24, 48 h of inoculation, data are means ± SD (*n* = 3), with statistically significant differences (*p <* 0.05).

Total phenolic compounds were determined at intervals 2, 24 and 48 h in tomato leaves after infection by *A. cerealis* and treated with *T. harzianum*. The total phenolic content in tomato plant treated with the pathogen was significantly increased in all treatment periods especially after two hours by 104.8 ± 0.51 mg/g FW, whereas plants treated with *T. harzianum* only did not induce the same response and the total phenolic compounds decreased. The phenolic content was significantly increased in the plant infected with *A. cerealis* and treated *T. harzianum* (Figure 6). The results indicated that the biological control stimulates production of the total phenolic compounds compared with the case with the pathogen alone or the saprophytic fungus alone.

### 2.5. Effect of Infection Period on the Total Flavonoid Production of Tomato Leaves during the Biological Control

**Figure 7:**
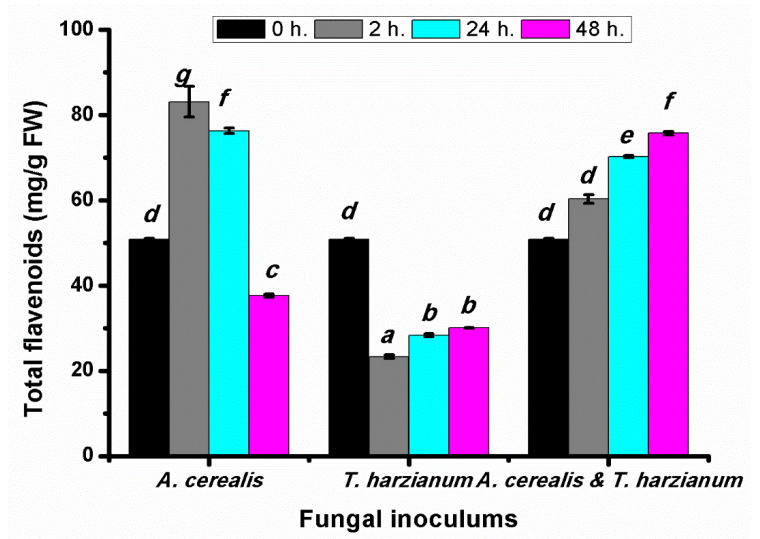
Total flavonoids (mg/g FW) of tomato leaves (*S. lycopersicum* L.) under the effect of pathogen (*Alternaria cerealis* MT808477), and pathogen inhibitor (*Trichoderma harizainum*) after 0, 2, 24, 48 h of inoculation, data are means ± SD (*n* = 3), with statistically significant differences (*p <* 0.05).

The flavonoid contents of infected tomato leaves were determined after different *A.* *cerealis* infection periods (2, 24, and 48 h) to clarify the damage caused by the pathogen. The data in Figure 7 show that the total flavonoid contents in tomato leaves significantly increased after 2 and 24 h by the biotic elicitor (*A.* *cerealis*) infection. This behavior is related to the antibiotic stress defense strategy of plant cells, which may involve increased flavonoid production to reduce the accumulation of reactive oxygen species in response to stress. Total flavonoid contents decreased after 48 h, likely because of the toxic effect of ROS accumulation on the plant cells, whereas the total flavonoids content in tomato plants treated with *T. harzianum* alone was significantly decreased in all incubation periods. One way ANOVA (*p* < 0.05) highlighted that the highest values of total flavonoids content were found in plant treatment with *A. cerealis* and *T. harzianum* by 75.76 ± 1.2 mg/g FW after 48 h.

### 2.6. Effect of Infection Period on the Total Terpenoids Production of Tomato Leaves during the Biological Control

**Figure 8:**
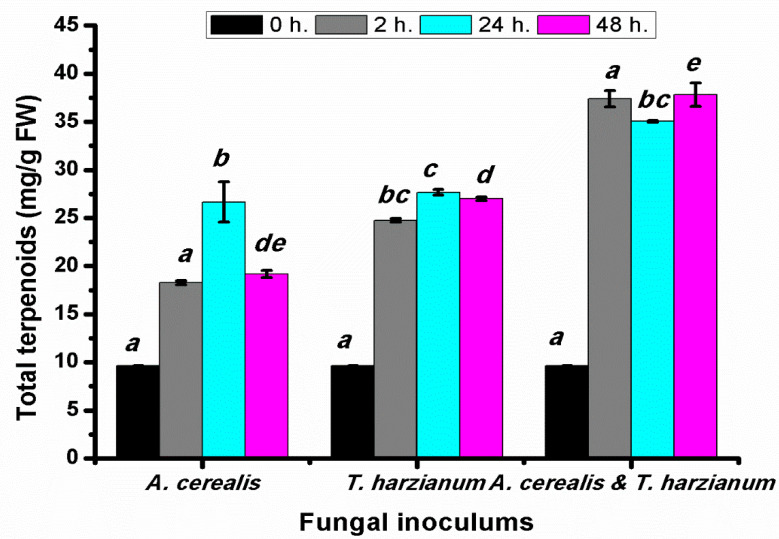
Total terpenoids (mg/g FW) of tomato leaves (*S. lycopersicum* L.) under the effect of pathogen (*Alternaria cerealis* MT808477), and pathogen inhibitor (*Trichoderma harizainum*) after 0, 2, 24, 48 h of inoculation, data are means ± SD (*n* = 3), with statistically significant differences (*p <* 0.05).

Total terpenoids were determined after 2, 24, and 48 h of the biological control of *Alternaria* *cerealis* by *T. harzianum*. Data shown in Figure 8 clarify that the total terpenoid content in infected tomato plants with *A.* *cerealis* was significantly increased after all incubation, especially after 24 h. Additionally, plants treated with *T. harzianum* induced the response of total terpenoids in all treatments. In the same manner, combination of *A. cerealis* and *T. harzianum* stimulates production of the terpenoid contents, compared with the case with the pathogen alone or the saprophytic fungus alone. The highest terpenoid content was recorded after 48 h by 37.83 ± 1.2 mg/g FW.

### 2.7. Effect of Infection Period on the Total Antioxidant Contents of Production Tomato Leaves during the Biological Control

**Figure 9:**
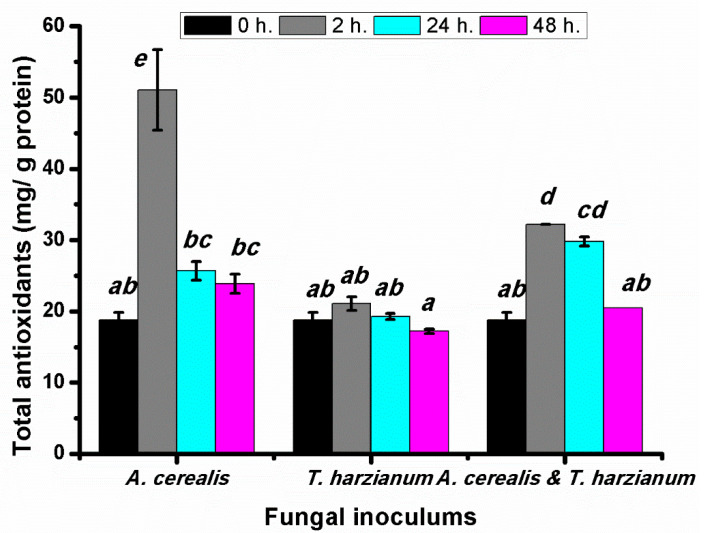
Total antioxidants (mg/g protein) of tomato leaves (*S. lycopersicum* L.) under the effect of pathogen (*Alternaria cerealis* MT808477), and pathogen inhibitor (*Trichoderma harizainum*) after 0, 2, 24, 48 h of inoculation, data are means ± SD (*n* = 3), with statistically significant differences (*p <* 0.05).

The total antioxidant contents of infected tomato leaves were determined after different *A.* *cerealis* infection periods (0, 2, 24, and 48 h.). Total antioxidant determination is a rapid and easy method of measuring the antioxidant pool under biotic stress, as illustrated in Figure 9. Total the total antioxidant content in tomato plants infected with *A.* *cerealis* after two hours (51.05 ± 5.64 mg/g protein). Then, a decrease in total antioxidant was observed after the remaining incubation periods (24 and 48 h), which could be attributed to the toxic effects of ROS accumulation on plant cells. Results additionally explained that the total antioxidants in tomato plants treated with *T. harzianum* were significantly increased specially after two hours. On the other hand, the tomato plant infected with *A. cerealis* and treated with *T. harzianum* showed increase in total antioxidant contents after two hours.

### 2.8. Effect of Infection Period on the Malondialdehyde Production of Tomato Leaves during the Biological Control

**Figure 10:**
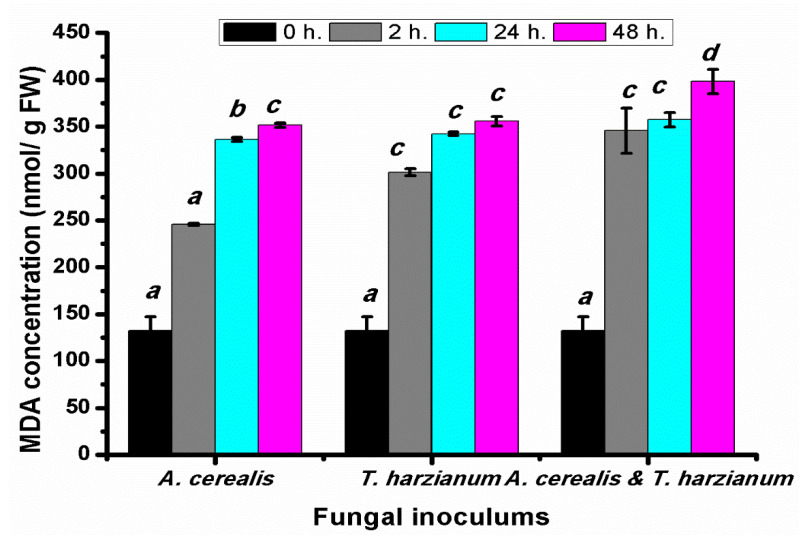
MDA (nmol/ g FW) of tomato leaves (*S. lycopersicum* L.) under the effect of pathogen (*Alternaria cerealis* MT808477), and pathogen inhibitor (*Trichoderma harizainum*) after 0, 2, 24, 48 h of inoculation, data are means ± SD (*n* = 3), with statistically significant differences (*p <* 0.05).

The *Malondialdehyde* (MDA) contents of tomato leaves were determined after different *A.* *cerealis* infection periods (0, 2, 24, and 48 h). The content of MDA, a product of polyunsaturated fatty acid peroxidation in cells, was determined in tomato plants exposed to the fungus to assess the degree of membrane damage resulting from *A.* *cerealis* infection. MDA contents was significantly increased in infected plants or treated with *T. harzianum* or both, and it dramatically increased with increasing the incubation periods. The highest content of MDA was recorded in the plant infected with *A. cerealis* by 245.70 ± 1.14 mg/g FW after 2 h, 336.47 ± 2.09 mg/g FW after 24 h, and 351.47 ± 2.56 mg/g FW 48 h. It was worth to mentioning that the correlation between MDA content and fungal filtrate was positive. The highest concentration of MDA was found in the plant treated with *T. harzianum* and *A. cerealis* after 48 h, as revealed in Figure 10.

### 2.9. Effect of Infection Period on Catalases (CAT) and Peroxidase (POX) Antioxidant during the Biological Control

**Figure 11:**
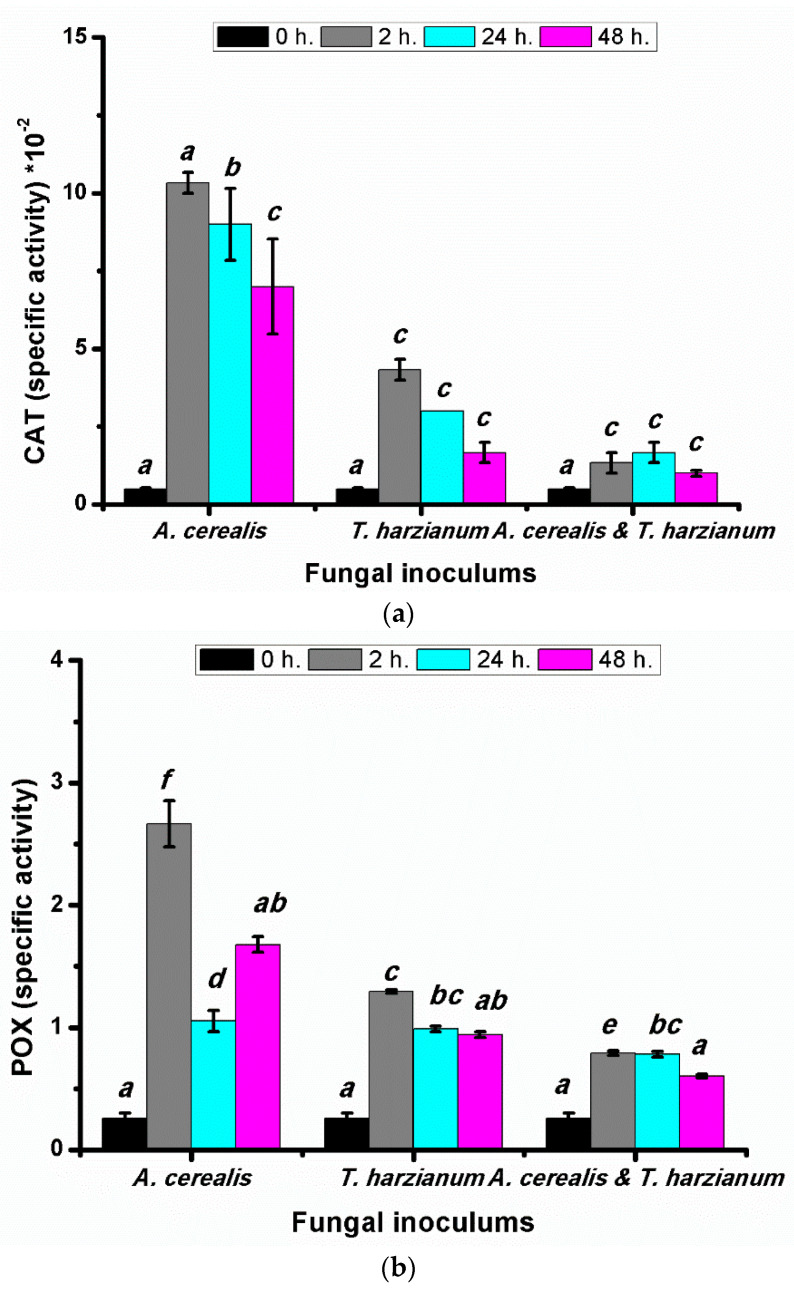
CAT (**a**) and POX (**b**) (specific activity) of tomato leaves (*S. lycopersicum* L.) under the effect of pathogen (*Alternaria cerealis* MT808477), and pathogen inhibitor (*Trichoderma harizainum*) after 0, 2, 24, 48 h of inoculation, data are means ± SD (*n* = 3), with statistically significant differences (*p <* 0.05).

Enzymatic antioxidants are the first line of defense against fungal pathogens. Catalase catalyzes the decomposition of H_2_O_2_ into water and oxygen to decrease the toxic effect of the stress. In our study, in tomato plants treated with the *A.* *cerealis* CAT activity was highest after two hours (0.103 ± 0.003 mg protein^−1^ min^−1^) and then decreased after 24 h (Figure 11a), whereas the plants treated with the *T. harzianum*, the CAT-specific activity was drastically decreased with increasing incubation periods. In the biological control treatment (*Alternaria* *cerealis* and *T. harzianum*), the highest CAT-specific activity was recorded after 24 h. The obtained data demonstrated that, in tomato plants treated with *Alternaria* *cerealis* and *T. harzianum*, there were adverse effects on the peroxidase enzyme (POX) activity (Figure 11b). The highest activity was observed after two hours in plant samples that were infected with *A.* *cerealis* (2.67 ± 0.19 mg protein^−1^ min^−1^), whereas the POX activity in plants treated with the *T. harzianum* was progressively decreased with increasing incubation periods after 48. The treatment with *A. cerealis* and *T. harzianum* displayed significant increase after 2 and 24 h in tomato plants.

### 2.10. Antimicrobial Activity

**Figure 12:**
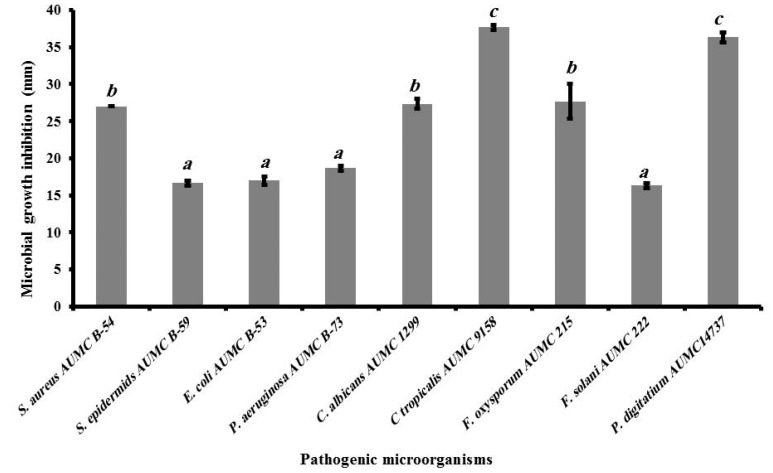
The antimicrobial activities of extract of infected tomato plant as microbial growth inhibition zone (mm) against Gram negative bacteria (*Escherichia coli* AUMC B-53 and *Pseudomonus aeruginosa* AUMC B-73), Gram positive bacteria (*Staphylococcus aureus* AUMC B-54 and *Staphylococcus epidermids* AUMC B-59), yeasts (*Candida albicans* AUMC 1299 and *Candida tropicalis* AUMC 9158) and filamentous fungi (*Fusarium oxysporum* AUMC 215, *Fusarium solani* AUMC 222, *Penicillium digitatium* AUMC14737), data are means ± SD (*n* = 3), with statistically significant differences (*p <* 0.05).

**Figure 13:**
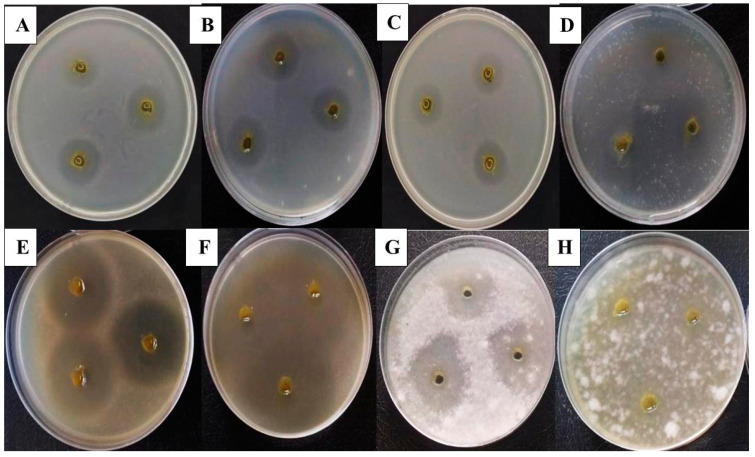
Microbial growth inhibition using infected tomato leaves (*S. lycopersicum* L.) by *Alternaria cerealis* (MT808477) against Gram negative bacteria (*Escherichia coli* AUMC B-53 (**A**) and *Pseudomonus aeruginosa* AUMC B-73 (**B**)), Gram positive bacteria (*Staphylococcus aureus* AUMC B-54 (**C**) and *Staphylococcus epidermids* AUMC B-59 (**D**)), yeasts (*Candida albicans* AUMC 1299 (**E**) and *Candida tropicalis* AUMC 9158 (**F**)), and filamentous fungi (*Fusarium oxysporum* AUMC 215 (**G**) and *Fusarium solani* AUMC 222 (**H**)).

The extract of infected tomato plants inhibited the growth of nine plant and human pathogenic microorganisms, namely *E. coli* AUMC B-53 (A), *P. aeruginosa* AUMC B-73 (B), *S. aureus* AUMC B-54 (C), *S. epidermidis* AUMC B-59 (D), *C. albicans* AUMC 1299 (E), *C.* *tropicalis* AUMC 9158 (F), *F. oxysporum* AUMC 215 (G), *F. solani* AUMC 222 (H), and *P. digitatum* AUMC14737, as illustrated in Figure 12 and Figure 13. The inhibition zones obtained ranged between 16.3 and 36.3 mm, and the highest antibacterial activities were recorded against *S. aureus* (27 ± 0 mm), *P. aeruginosa* (18.67 ± 0.33 mm), and *E. coli* (17 ± 0.58 mm). *S. epidermids* was also affected by the ethanolic extracts and showed an inhibition zone of 16.67 ± 0.33 mm. The phytoalexin extract demonstrated greater antifungal activity against *C. tropicalis* (37.67 ± 0.33 mm) than against *C. albicans* (27.33 ± 0.67 mm). The extract of infected plants also showed the greatest antifungal activity against *P. digitatium* (36.3 ± 0.67 mm), followed by *F. oxysporum* (27.7 ± 2.33 mm) and *F. solani* (16.3 ± 0.33 mm).

## 3. Discussion

Tomato plants represent a rich source of minerals, vitamins and the antioxidant lycopene, contributing to a healthy and balanced diet [16]. *Alternaria* is the common pathogen of leaf spots that cause diseases in tomato plants. It is the first record of *Alternaria* *cerealis* as a tomato phytopathogen that causes dark spot lesions. In this respect, Akhtar et al. [17] isolated 35 strains of *Alternata* sp. from rotten fruits in the fields and markets, and observed that only one isolate was able to produce symptoms of leaf blight and was the first to report blight in Pakistan. Additionally, in Korea, leaf spots disease was first recorded in sesame plants (*Sesamum indicum* L.) caused by *A. simsimi* [18]. Fungal diseases are the primary limiting factor during the commercial production of tomato plants in greenhouses. Infection by these pathogens can result in plant death and reduced yields. Many management approaches have been taken to prevent the establishment of diseases and to minimize the development of pathogens in tomato crops. These include the development of resistant or tolerant cultivars and the use of biological control agents. In recent years, the mechanisms that may contribute to the control of fungal diseases include induction of plant resistance, competition with other microbes and production of inhibitory chemicals [19]. The presence of *A. cerealis* MT808477 in tomato plants should be considered as the cause of food disqualification. In order to reduce the incapacity and loss in tomato plant production caused by this pathogen, biological control agent was used to controlling the leaf spot disease caused by *A. cerealis*.

Biological control aims to decrease the destruction caused by phytopathogens by using biotic agents, which control the oxidative stress in infected plants by up-regulating cell metabolites and antioxidant enzymes such as peroxidase, catalase, and superoxide dismutase [20]. *Trichoderma* can promote plant growth and induce plant defense responses to different biotic stress [5,21]. The application of *T. harzianum* in biological control showed a significant reduction in disease incidence, with some evidence supporting its ability to induce defense mechanisms in plants [21,22]. The phenolic compounds, total flavonoids and total antioxidant contents were significantly increased in a plant infected with *A.* *cerealis* after two hours, whereas the total terpenoid was significantly increased after 24 h but decreased after 2 and 48 h at the same level. CAT and POX activities were increased after 2 h, but in the case of using *Trichoderma* only, the total phenolic and flavonoids contents in tomato saplings did not induce the in treatments. Total antioxidants, CAT and POX activities were significantly increased after 2 h but, the total terpenoid content was significantly increased after 24 h, whereas the highest of MDA were recorded values after 48 h. On the other hand, under the combined stress of *A. cerealis* and *T. harzianum* in tomato plants, the results indicated that the production of the total phenolic compounds, flavonoids, MDA and terpenoid were significantly increased with an increased infection period compared with the case with the pathogen alone or the saprophytic fungus alone. The highest contents were observed after 48 h, while the total antioxidant contents were significantly increased after two hours. In this respect, Ramamoorthy et al. [23] mentioned the accumulation of phenolic compounds and PR-Proteins which have contributed to the restriction of invasion of *Fusarium oxyporum* f. sp. *lycopersici* in tomato roots. Similar results are reported by El-Khallal [24], who showed an increase in phenolic compounds, CAT and POX in infected tomato plants with *Fusarium oxyporum*. Flavonoids play an important role in controlling the response of plants to biotic stresses [25,26]. The present results are similar to the finding of Matta [27] who reported that infection of tomato leaves with different forms of *Fusarium oxysporum* increased the levels of phenols and flavonoids content. Additionally, Abdel-Monaim [28] observed an increase in phenolic compounds and total flavonoid in *Faba bean* plants inoculated with *Fusarium oxysporum*.

The current results indicated that the total antioxidant content was increased in tomato plants infected with *A.* *cerealis* after two hours. Then, a decrease in total antioxidant was observed after the remaining incubation periods (24 and 48 h). In this respect, Zehra et al. [29] found that the activity of antioxidant enzymes was increased in tomato plants infected with *Fusarium oxysporum* f. sp. *lycopersici* at 24 h, peaking at 72 h and was decreased slightly in all applied treatments compared to the control. The results clarify that the total terpenoid content in infected tomato plants with *A. cerealis* was significantly increased after 24 h. This result is in harmony with the findings of Zehra et al. [29] who stated that the activity of CAT and POD were significantly increased at 48 in comparison to control in the tomato plants infected with *F. oxysporum* f. sp. *lycopersici*. Zhou et al. [30] found that significantly decreased of CAT activity and increased POX in both tomato cultivars at drought and heat stresses after third day. The increase in the POX activity in tomato plants in response to infection with *Botrytis cinerea* infection could confirm earlier reports of Kużniak and Skłodowska [31]. Hassan et al. [32] recorded low percentages of chocolate spot disease severity and the highest levels of peroxidase activities. The results indicated that the MDA content was significantly increased in infected plants. In this instance, Zehra et al. [29] showed that the MDA was significantly higher in tomato plants infected with *Fusarium oxysporum* f. sp. *lycopersici.* In oxidative stress, total soluble protein and MDA were usually studied as an indicator of metabolic changes, because under stress conditions, ROS can cause severe damage by interacting with cellular components (such as proteins, nucleic acids, and lipids) [33].

Treatments with *Trichoderma* strains exhibited a slight increase of antioxidant activity in strawberry fruits [34]. Wang et al. [35] reported that *Trichoderma aspereullm* induced the activity of ROS scavenging enzymes in plants kept at a high level after inoculation, which may minimize the damage to the plants during the colonization of *T. aspereullm*. Our results are greatly similar to the results obtained by Zhang et al. [36] who found that the use of *Trichoderma longibrachiatum* increased POX and CAT activities in tomato plants. In this respect, Dini et al. [37] reported that *Trichoderma* increased the phenolic compounds and antioxidant potential in olive leaf samples. Additionally, Youssef et al. [38] observed that the disease symptoms of plants treated with biological control agents were reduced by 71.7% compared with plants infected with *R. solani*, while the disease incidence rate of tomato seedlings treated with biological control agents was only 10.33%. Presently, various reports indicate that *Trichoderma* induces systemic resistance by releasing not only proteins, but also secondary metabolites [39]. *Trichoderma* species can induce profound impacts or changes in different species of plant gene expression under biotic stress [40]. These results are in agreement with those obtained by EL-Tanany et al. [41]. They reported an increase in phenolic compounds and total flavonoids content in infected tomato plants with *Alternaria solani* after being treated with *T. viride* or *T. hamatum***.** This result is similar to the finding of Mayo-Prieto et al. [42] who found that the presence of *T. velutinum* and *R. solani* increased the production of terpenoid content in bean leaves compared to the control. In this respect, the soluble protein levels induced by *T. aspereullm* may indicate strong tolerance to osmotic stress in the early stage after inoculation [35]. The highest concentrations of MDA and H_2_O_2_ were found in the plant treated with *T. harzianum* and *A. cerealis*. Our results are similar to the results obtained by Zehra et al. [29] who recorded that the maximum MDA production in tomato plants which were treated with salicylic acid along with pathogen or in combination with *T. harzianum*, while minimum production was shown by the *Fusarium oxysporum* f. sp. *Lycopersici* + *T. harzianum* + methyl jasmonate treated plants. In accordance with this result, Youssef et al. [38] showed that increase in the enzymatic activities in plants treated with *Rhizoctonia solani* and decreased significantly in tomato plants pretreated with a combination of *T. harzianum* and *R. solani*.

Plants producing antibiotic compounds against a wide variety of pathogenic microorganisms, including phenolic acids and polyphenols, flavonoids, and terpenoids have been described [11,12]. The antibacterial and antifungal activities of extracted tomato plants are linked to its chemical composition, and to the functional groups of the major compounds (flavonoids, phenols, terpenes compounds and chlorogenic acid, caffeic acid) [43,44]. Additionally, tomato leaves contain more flavonoids, solavetivone, lubimin, phytuberin, phytuberol, rishitin and glutinosone that have antimicrobial agents and tare toxic, acting as a defense compound against a wide range of pathogens and pests. This indicates that these compounds can be used as natural therapeutic agents [44]. The current results indicated that the extracts of infected tomato plants after being infected with *A.* *cerealis* have more antimicrobial activity, as indicated by the inhibition zones obtained ranged between 16.3 and 36.3 mm. This observation agrees with that of Kim et al. [44], who reported that acetonic extract from tomato leaves inhibited *Fusarium oxysporum* f. sp. *lycopersici*, *Glomerella cingulata*, and *Rhizoctonia solani*. In accordance, tomato plant extracts inhibited the growth of pathogens such as *E. coli*, *Salmonella Typhimurium*, *Staphylococcus aureus*, and *Listeria ivanovii*, yielding an inhibition zone of 8.0 to 12.9 mm in diameter [45].

## 4. Materials and Methods

### 4.1. Pathogen Isolation

Fresh tomato leaves (*Solanum lycopersicum* L.) with dark spot lesions were collected from agriculture in a field (Lat. 27°11′16″ N, 31°10′13″ E) and examined under a light microscope to confirm fungal infection. *Alternaria* was isolated on potato dextrose agar medium (PDA) at 28 °C ± 1 °C according to the method of Meena et al. [46].

### 4.2. Phytopathogenic Ability

The pathogenic ability of *A.* *cerealis* was confirmed as follows. Pathogen inocula were prepared by collecting fungal spores from the PDA plates, suspending the same in sterile potato dextrose broth fortified with 0.1% Tween-80, and diluting the solution to a concentration of 1 × 10^5^ conidia/mL. The inocula were sprayed on four-week-old tomato seedlings. The development of raised dark spot lesions on the green leaves demonstrated successful infection. Non-sprayed tomato seedlings were used as healthy plants (control), as described by Blagojević et al. [47].

### 4.3. Tissue Processing for Light Microscopy

Transverse sections of healthy tomato leaves and *A.* *cerealis* dark spot lesions were obtained and examined under a light microscope (Olympus CX41 Plan CN, Japan). The specimens were collected from infected and uninfected tomato leaves, dissected using a rotary microtome into sections measuring 10–20 μm in thickness, and then immediately fixed in formalin/acetic acid/alcohol (*v:v*, 5/5/90). The leaves were immersed in light green stain, mounted in 1% glycerin, and covered. The coverslip was sealed with DPX. Finally, the slides were examined under a light microscope and photographed [48].

### 4.4. Scanning Electron Microscopy

Healthy and infected tomato leaf surfaces were analyzed by scanning electron microscopy (SEM) to observe the characteristics of pathogen infection. The plant leaves were immersed in 0.1 M sodium cacodylate buffer and 2.5% pure glutaraldehyde, stored at 4 °C for 6 h, and then soaked in 1% osmium tetroxide for 2 h. The samples were then dehydrated in a series of ethanol solutions with increasing concentration from 50% to 100%, dried using a Polaron device with Freon 13, and coated with gold by ion sputtering (JEOL-1100 E, Japan). The samples were examined and photographed under a scanning electron microscope (JSM 5400 LV; JEOL, Japan) [49].

### 4.5. Alternaria cerealis Morphological Identification

*A.cerealis* (E.G. Simmons & C.F. Hill) was identified according to its morphological properties, including colony morphology, color, conidia, conidiophore, and chlamydospore formation, by using a light microscope as described by Simmons [50].

### 4.6. Alternaria cerealis Genetic Identification

#### 4.6.1. DNA Extraction

A small portion from the fungal growth of 7-day-old culture of *Alternaria* sp. AUMC 14484 grown on PDA plates at 30 ± 1 °C were collected and transferred into an Eppendorf tube containing 800 μL CTAB buffer (3% CTAB, 1.4 M NaCl, 0.2% Mercaptoethanol, 20 mM EDTA, 100 mM TRIS-HCl pH 8.0 and 1% PVP-40), incubated at 65 °C for 30 min. This was mixed with chloroform and isoamyl alcohol, and centrifuge at 10,000× *g* for 10 min. For DNA precipitation precooled isopropanol was added, incubate at 4 °C overnight, and centrifugation at 13,000× *g* for 10 min. The pellets were washed with 200 μL washing buffer (76% ethanol and 10 mM ammonium acetate) then 200 μL TE buffer supplemented with 10 mg/mL RNase were add. After incubation at 37 °C for 30 min, 100 μL of 7.5 M ammonium acetate and 750 μL ethanol were added, then centrifuged at 13,000× *g* for 10 min at room temperature. The supernatants were completely discarded and the pellets were suspended, individually in 100 µL sterile distilled water.

#### 4.6.2. PCR and Sequencing of ITS

The universal primers ITS1 and ITS4 [51] were used for amplification of the internal transcribed spacer (ITS) region (SolGent, Daejeon, Korea). Amplification was conducted using the following PCR conditions: denaturation at 95 °C for 15 min (one cycle), denaturation at 95 °C for 20 s (30 cycles) then annealing at 50 °C for 40 s and extension at 72 °C for 1 min (30 cycles), with a final extension step of 72 °C for 5 min. The purified PCR products were confirmed on 1% agarose gel by electrophoreses, then eluted and sequenced in the forward and reverse directions using the same primers (ITS1 and ITS4) and the incorporation of ddNTP in the reaction mixture [52].

#### 4.6.3. Alignments and Phylogenetic Analyses

Sequences of the nearest closely related species belonging to genus *Alternaria* were downloaded from GenBank including sequences of the type specimens. ITS sequences of *Alternaria* sp. AUMC 14484 in this analysis was uploaded to GenBank as MT808477. Sequences of *Alternaria* sp. AUMC 14484 in the present study and those retrieved from GenBank were aligned together using MAFFT (version 6.861b) with the default options. Alignment gaps and parsimony uninformative characters were treated by BMGE. Maximum (ML) likelihood and Maximum (MP) parsimony analyses conducted via PhyML 3.0. The best optimal model of nucleotide substitution for the ML analyses was determined using Smart Model Selection (SMS) version 1.8.1. The phylogenetic tree was visualized using FigTree version 1.4.3 [53].

### 4.7. Inhibition of A. cerealis (MT808477) Using Saprophytic Trichoderma harzianum

One-week-old *A. cerealis* spores grown on PDA medium at 28 ± 2 °C were scratched, suspended in 0.1% Tween-80/sterilized distilled water, and diluted to 10^5^ conidia/mL. One milliliter of aliquot spore suspension of *A. cerealis* was transferred directly into empty sterilized plates, after which about 15 mL of sterilized PDA medium cooled to just above solidifying temperature was added to the plates. The plates were rotated by hand in a broad swirling motion to disperse the spore suspension in the medium. After solidification, *T. harzianum* was inoculated in the center of the plates, which were incubated at 28 ± 2 °C for 1 week.

### 4.8. Greenhouse Experiment

#### 4.8.1. Soil Accommodation

A mixed sandy loam soil collected from soil layers until 25 cm depth from the Assiut University farm. Physico-chemically soil properties were assessed including in ppm: sodium 186.3, potassium 46.8, calcium 574, chloride 887.5, magnesium 344.4, and carbonate 1525. The soil was dried, sieved by 2 mm sieve pores, and sterilized at 121 °C for 20 min in steam autoclave three times. Then, the soil was transferred to 30 cm plastic pots for planting.

#### 4.8.2. Experimental Design

A greenhouse experiment was carried out in a completely randomized design, and four replicates. Thirteen-day-old tomato plants kindly obtained from the Agriculture Research Center were utilized for this experiment. Soil pots were transferred into greenhouse at average max. temperature 35 °C ± 2 °C, and average max. temperature 22 °C ± 2 °C, relative humidity 42%–55%, and a photoperiod of 14 h. In the inocula, *A. cerealis* was included at a concentration of 10^5^ conidia/mL. *T. harzianum* filtrate was obtained by transferring one disk (7 mm) of PDA medium covered with 3-day-old mycelia in 100 mL of potato dextrose broth, which was incubated at 28 °C ± 2 °C for 7 days. Flasks were filtered to separate and discard the mycelial mass. *T. harzianum* was applied on *S. lycopersicum* plants infected with *A. cerealis*. The filtrate sprayed after injecting tomato plants with *A.* *cerealis* conidial suspension (10^5^ conidia/mL) [54]. Plant leaves were collected after 0, 2, 24, and 48 h for physiological assays and the plant were examined every 2 days until two weeks for any symptoms appearing.

### 4.9. Physiological Analysis of Plants

#### 4.9.1. Determination of Total Phenol

The Folin–Ciocalteu method described by Meda et al. [55] was used to determine the concentration of total phenol content. The tomato leaves were dissolved in methanol (1 mL). Mix 100 µL of extract with 750 µL of 1N Folin-Ciocolteu reagent (1:10). After standing at room temperature for 5 min, 60 µL Na_2_CO_3_ (7.5%) was added to the extract. The mixture was incubated for 30 min at room temperature. The absorbance of the reaction mixture was measured at 750 nm. The phenol concentration in the extract was determined from a standard curve prepared from gallic acid and expressed as µg/g fresh weight.

#### 4.9.2. Total Flavonoid Determination

The flavonoid contents of the tomato leaves were determined according to the method of Chang et al. [56] using aluminum chloride reagent. The absorbance of the leaf extracts was read at 496 nm and expressed as milligrams of quercetin equivalents.

#### 4.9.3. Total Terpenoid Determination

The determination of total terpenoid content was carried out by a colorimetric method in accordance with the process reported by Fan and He [57]. One gram of leaves was dissolved in 1 mL of dichloromethane. The extract was mixed with a solution of 150 μg of vanillin dissolved in 5% glacial acetic acid *w/v* and heated at 60 °C for 45 min, followed by cooling in ice-water. Terpenoid content at 548 nm was expressed as milligram ursolic acid equivalents (mg ursolic acid/g extract).

#### 4.9.4. Total Antioxidant Determination

Total nonenzymatic antioxidant activity was measured at 695 nm and expressed in micrograms per gram of protein using sulfuric acid–ammonium molybdate reagent as described by Prieto et al. [58].

#### 4.9.5. Determination of Malondialdehyde (MDA)

The leaf material (0.5 g) was homogenized in 5 mL of ethanol and then centrifuged at 10,000 rpm for 5 min. The supernatant was added to 0.5% thiobarbituric acid in 20% trichloroacetic acid. The concentration of lipid peroxidation (MDA) was quantified from the extinction coefficient of 155 mM^−1^ cm^−1^. The result was expressed as nmol g^−1^ fresh weight (FW) [59].

#### 4.9.6. Determination of Catalases (CAT) and Peroxidase (POX) Antioxidant

Frozen leaf segments (1 g) were ground to a fine powder in liquid nitrogen and then homogenized in 100 mM potassium phosphate buffer containing Na_2_-EDTA and polyvinylpyrrolidone. The supernatant was collected and used for evaluating the activities of all tested enzymes. Protein content was assayed by the method of Lowry et al. [60], using bovine serum albumin as a standard. Catalase (CAT) activity was determined by a reaction in a medium containing 50 mM potassium phosphate buffer, H_2_O_2_ (10 mM), and the enzyme, by measuring the decrease in absorbance at 240 nm (DA_240_ mg protein^−1^ min^−1^) [61]. Peroxidase (POX) activity was estimated by mixing the enzyme with potassium phosphate buffer containing H_2_O_2_ (6.5 mM) and guaiacol (1.5 mM). The data were expressed as DA_470_ mg protein^−1^ min^−1^ [62].

### 4.10. Antimicrobial Activity

The antimicrobial activities of the infected tomato leaves were tested against nine plant and human pathogenic microorganisms, including Gram-negative bacteria (i.e., *Escherichia coli* AUMC B-53 and *Pseudomonus aeruginosa* AUMC B-73), Gram-positive bacteria (i.e., *Staphylococcus aureus* AUMC B-54 and *Staphylococcus epidermids* AUMC B-59), yeasts (i.e., *Candida albicans* AUMC 1299 and *Candida tropicalis* AUMC 9158), and filamentous fungi (i.e., *Fusarium oxysporum* AUMC 215, *Fusarium solani* AUMC 222, and *Penicillium digitatium* AUMC14737) were evaluated. The microorganisms were obtained from the Center of Prof. A.H. Moubasher for Mycological Sciences, Assiut University, Egypt. Exactly 1 mL of the growing pathogens in broth medium was combined with 15 mL of nutrient agar medium for bacteria and Sabouraud dextrose agar medium for fungi, and the mixture was allowed to rest until complete solidification. Wells measuring 3 mm in diameter were cut from the agar plates, and 50 μL of the leaves extract was delivered into each well. The plates were incubated at 35°C for 24 h (for bacteria), 48 h (for yeasts), or 5 days (for filamentous fungi), and the antimicrobial activity of the extracts was evaluated by measuring the inhibition zone (in millimeters) produced against the tested pathogens.

### 4.11. Statistical Analysis

Statistical analysis was conducted using one way analysis of variance (ANOVA), and differences among means were determined at *p* ≤ 0.05 by using Duncan’s multiple range tests. SPSS software version 25 was for all statistical calculations.

## 5. Conclusions

Tomato phytopathogen *A. cerealis* MT808477 is controlled effectively by a saprophytic fungus *T.* *harzianum.* The biological control process suppresses efficiently the plant disease through the induction of the plants defense. Biological control represents a safe and effective solution to phytopathogens that decreases plant cell stress by stimulating various defensive agents like phenol, flavonoids, terpenoids, antioxidant, malondialdehyde and the antioxidant enzymes with less harmful effects on the plant cells themselves. These active compounds could be utilized in biotechnological applications as valuable antimicrobial and therapeutic agents.

## Data Availability

The following information was supplied regarding data availability: fungal sequencing data are deposited in the NCBI (http://www.ncbi.nlm.nih.gov) web site under accession numbers; *Alternaria cerealis* (AUMC 14484) MT808477.
